# Palliative surgery for primary sarcoma in the abdominal aorta: A case report and review of the literature

**DOI:** 10.3892/ol.2013.1594

**Published:** 2013-09-25

**Authors:** JU-LIANG ZHANG, SU-MIN YANG, QING YAO, JIANG-HAO CHEN, TING WANG, HUI WANG, JING FAN, RUI LING, JUN YI, SHI-FANG YUAN, LING WANG

**Affiliations:** 1 Department of Vascular and Endocrine Surgery, Xijing Hospital, Fourth Military Medical University, Xi’an, Shaanxi 710032, P.R. China; 2 Department of Cardiac Surgery, Affiliated Hospital of Medical College, Qingdao University, Qingdao, Shandong 266003, P.R. China

**Keywords:** abdominal aortic aneurysm, palliative surgery

## Abstract

Primary sarcoma of the aorta is extremely rare and accounts for <1% of all sarcomas. The present study describes the case of a 45-year-old male who presented with lower limb and abdominal pain. Abdominal computed tomography (CT) and magnetic resonance (MR) arteriography revealed a tumor that extended from the infrarenal aorta to the aortic bifurcation. The external and internal iliac arteries were occluded by the tumor incursion. Palliative surgery was performed for the sarcoma since the patient refused a radical resection. To improve the blood supply to the lower limbs, an axillary bifemoral bypass was established. Following the surgery, the pain was significantly reduced. However, the patient succumbed due to extensive metastasis 6 months after this surgery. Aortic sarcoma is an extremely rare disease with a poor prognosis. A diagnosis at a relatively early stage is necessary for a longer survival time. Radical surgery is the most significant treatment. Patients at advanced stages should consider palliative surgery in order to improve their quality of life.

## Introduction

Primary sarcoma of the aorta is extremely rare and accounts for <1% of all sarcomas (1). A prompt diagnosis prior to organ ischemia and systemic metastasis is difficult due to the various clinical presentations of this disease. Despite the availability of various imaging studies, including computed tomography (CT) and magnetic resonance (MR) imaging, the condition may be mistaken for an aortic aneurysm or other arterial occlusive diseases (2,3). Treating a primary sarcoma of the aorta is also difficult since this disease is usually diagnosed at a relatively late stage. The present study describes a case of primary sarcoma in the infrarenal aorta and its palliative treatment. Written informed consent was obtained from the patient’s family.

## Case report

**Figure 1:**
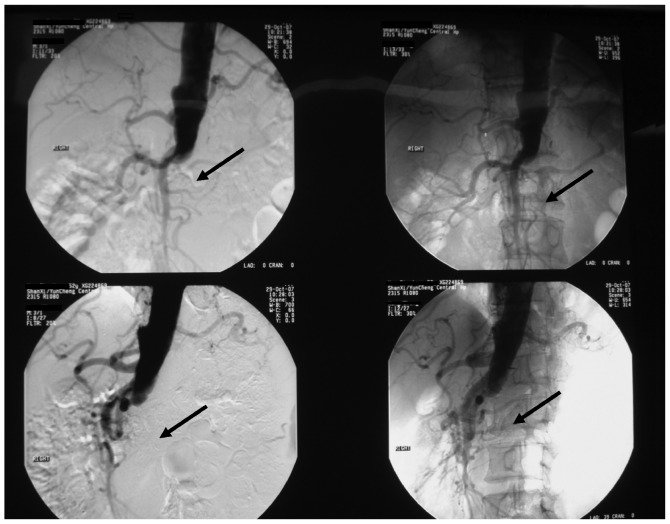
Angiography prior to surgery. The arrow indicates the aorto-iliac occlusion.

A 50-year-old male was initially admitted to a community hospital due to claudication, and was consequently diagnosed with arteriosclerosis obliterans. The vasodilator, cilostazol, was administered to the patient for ~1 month. However, the ischemia of the lower limbs worsened with significant pain on resting, as well as abdominal pain. Therefore, the patient was transferred to Xijing Hospital (Xi’an, Shaanxi, China). A physical examination revealed a pulsatile mass in the abdomen. The lower limbs were cold and the femoral, popliteal and dorsalis pedis arteries were pulseless. An abdominal CT scan demonstrated an irregular thrombus-like mass in the infrarenal aorta. MR arteriography revealed an almost complete occlusion of blood flow from the infrarenal segment of the abdominal aorta to the aortic bifurcation (Fig. 1).

**Figure 2:**
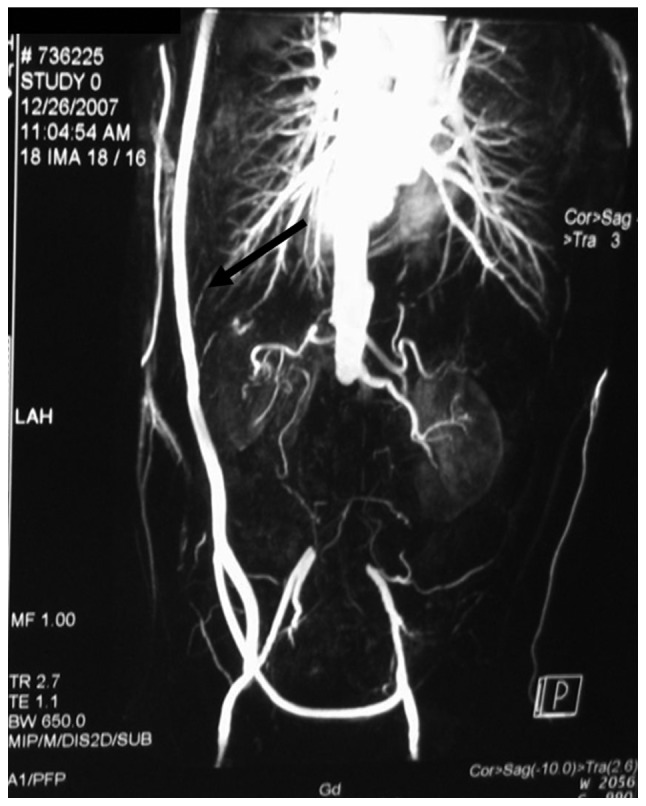
Angiography of the axillary bifemoral artery bypass. The arrow indicates the patent prosthetic bypass.

Initially, an abdominal aortic aneurysm (AAA) was diagnosed. Thus, an AAA resection and vascular reconstruction were planned. However, exposure of the abdominal aorta revealed the absence of thrombosis in the lumen. Instead, a tumor was identified covering the entire aortic wall. A section of the tumor was removed for frozen section analysis. The result suggested a sarcoma that originated from undifferentiated non-endothelial intimal stromal cells. Further exposure revealed that the tumor had invaded the vena cava. However, the patient refused radical surgery involving a major vascular resection and reconstruction due to the potential dangers. Therefore, a palliative treatment was decided upon. A large section of the tumor was removed and axillary bifemoral and femoro-femoral cross-over bypass grafts were established (Fig. 2).

The post-operative histology confirmed the diagnosis of a sarcoma. The lower limb pain improved significantly following the surgery and the patient was administered no other adjuvant therapies. At the 3-month follow-up appointment, the prosthetic bypass was patent, however, a local tumor had developed. The patient succumbed due to extensive metastasis 3 months later.

## Discussion

Aortic sarcoma is a rare disease. Aortic tumors are generally classified into two categories, the intimal-type and the mural-type, according to the site of the occurrence in the aortic wall. The intimal-type is a malignant mesenchymal tumor that is characterized by an intraluminal growth with tract obstruction and emboli seeding. The tumor grows in the direction of the aortic lumen, extending along the intima or growing as a polypoidal mass (4,5). Clinically, the primary presentations of a patient are symptoms of occlusion, including pulseless and painful extremities or abdominal pain, rather than symptoms that are directly associated with the primary tumor (4). The present case describes an intimal-type sarcoma. The post-operative histological examination identified spindle cells with various degrees of atypia overlying an acellular layer that contained collagen in the lumen of the involved artery. Immunohistochemical staining of the mesenchymal marker, vimentin, was positive.

The majority of intimal tumors are initially misdiagnosed as they have the same presentation as aorto-iliac occlusive or aneurysmal arteriosclerotic diseases (2,3). In the present study, the patient was first diagnosed with arteriosclerosis obliterans upon admittance to the community hospital. No further examinations were performed due to the lack of imaging instruments. When the patient was transferred to Xijing Hospital and correctly diagnosed, the tumor had already reached an advanced stage. In certain cases, hypertension is the primary manifestation due to renal artery stenosis or occlusion (3,6). Less commonly, patients may present with a ruptured aneurysm that is caused by the tumor (7). CT and angiography provide unique opportunities for the diagnosis and evaluation of aortic sarcomas. In the present case, CT with contrast enhancement had a limited role in the evaluation as the findings were suggestive of a thrombotic aneurysm. Compared with conventional angiography, MR angiography is more favored as it does not carry the risks of embolization or contrast-induced renal failure (8). A previous study has indicated that transesophageal echocardiography may also reveal an inhomogeneous and echodense mass with an outer membrane, which is unlike a thrombus and is suggestive of a primary aortic tumor (9).

The most effective treatments for an aortic sarcoma are radical resection and vascular reconstruction. The surgeries often present difficulties according to the tumor volume, depth of location and the proximity to the vital organs. For the patient in the present case, a major vascular resection and reconstruction was planned, as the aorta and the vena cava were involved. However, the patient refused radical resection due to the potential dangers. To improve the pain in the lower extremities, axillary bifemoral and femoro-femoral cross-over bypass surgeries were adopted. The result was satisfactory. When dealing with benign aortic occlusive disease, including abdominal aorta thrombotic aneurysms, ligation of the proximal neck of the aneurysm with an axillary bifemoral bypass graft may be considered. Certain studies of single patients or small collectives have reported that chemotherapy may benefit the overall survival of patients suffering from aortic sarcomas (1,4). However, sufficient data are lacking to provide evidence for this hypothesis (10).

In conclusion, aortic sarcoma is an extremely rare disease with a poor prognosis. A diagnosis at a relatively early stage is necessary for a longer survival time. Aortic sarcoma should be suspected in patients with symptoms of non-atherosclerotic-related aortic occlusive diseases or distal embolic events. Radical surgery is the most important treatment. For patients at advanced stages, palliative surgery may also be considered in order to improve their quality of life.
